# Towards improving community pharmacy-based mental health services in Nigeria

**DOI:** 10.1186/s40545-021-00316-9

**Published:** 2021-04-07

**Authors:** Adeboye Olakunle Bamgboye, Ibrahim Adebayo Hassan, Yusuff Adebayo Adebisi, Rachael Oluwatoyosi Farayola, Theogene Uwizeyimana

**Affiliations:** 1 Faculty of Pharmacy, Obafemi Awolowo University, Ile-Ife, Nigeria; 2 Bydow Pharmacy, Lagos, Nigeria; 3 Department of Pharmacy, Obafemi Awolowo University Teaching Hospitals Complex, Ile-Ife, Nigeria; 4 Faculty of Pharmacy, University of Ibadan, Ibadan, Nigeria; 5 Department of Public Health, Mount Kenya University Rwanda, Kigali, Rwanda

**Keywords:** Pharmacists, Community pharmacy, Mental health services, Nigeria

## Abstract

In Nigeria, there is a prevalence of aversive life circumstances that frequently assail the mental health and well-being of the citizens, mitigating the impact of which necessitates the institution of psychotherapy and other mental health care methods. These responsibilities, however, appear to be much more for pharmacists in low-resource settings where they are the most accessible healthcare professional. Some of these responsibilities include patient confidentiality as well as counseling patients on medication use, lifestyle as well as other personal matters that may arise in relation to their health. Mental health services including psychotherapy provide a range of therapeutic techniques that enable the patients (individual or groups) to develop effective coping strategies towards emotional and psychological difficulties, via methodic interactions with a mental health expert. In this commentary, we share suggestions on how to improve community pharmacy-based mental health services in Nigeria. With the expanding roles and responsibility for pharmacists beyond medication-related concerns comes the challenge of matching up the training of pharmacists with the broadening scope of practice in Nigeria. However, as pertinent as that might be, there are existing knowledge and competency gaps in keeping up with this trend. To correct these shortfalls, we contend that the training curricula for pharmacists in Nigeria be reviewed and/or expanded to provide adequate knowledge for pharmacy undergraduates and pharmacists about non-drug mental health care which will also impact psychotherapy services during their practice especially in the community settings.

## Commentary

There is a prevalence of aversive life circumstances in the society which frequently assail the mental health and well-being of Nigerians. Such negative circumstances include high rate of poverty, high unemployment rate, insecurity, traumatic experiences, human right abuse, and a deep-rooted belief in supernatural affliction, among others [1]. Thus, efforts to mitigate the impact of such factors and to promote or restore mental wellness have necessitated the institution of various mental healthcare methods [1]. There has been an increase in the day-to-day responsibilities of pharmacists the world over [2]. These responsibilities, however, appear to be much more for pharmacists in low-resource settings where they are the most accessible healthcare professional [3]. Some of these responsibilities include patient confidentiality as well as counseling patients on medication use, lifestyle as well as other personal matters that may arise in relation to their health. Mental health services including psychotherapy (or talk therapy) provide a range of therapeutic techniques that enable the patients (individual or groups) to develop effective coping strategies towards emotional and psychological difficulties, via methodic interactions with a mental health expert [4]. However, complicating the systemic cultural barriers towards the utility of psychotherapeutic interventions in Nigeria [4], mental health care is generally underfunded and with a significantly low psychiatrist–population ratio of about 1:1,000,000 [5]. To placate this, one of the groups of professionals that currently fill this mental health professional shortage gap for Nigerians are the community pharmacists. In this commentary, we share suggestions on how to improve pharmacy-based mental health services in Nigeria.

Community pharmacies are the first point of call for health services, in Nigeria, frequently more than the designated primary health centers. This is due to the ease of access, short waiting time, free consultation, and longer-term availability of the community pharmacists to the people [6]. For this position, pharmacists have been reinventing themselves to meet the dynamic health demands of the community [7]. One of such demands is the provision of mental health services for patients with such needs. These services range from counseling on traumatic life experiences to facilitating therapy for addiction and depressive disorders among others. In fact, Osemene and Erhun [7] underscored counseling as the most common self-reported participation in non-pharmacologic public health activities among community pharmacists. Also, given its strategic position, community pharmacy-based provision of behavioral activation therapy for the treatment of depression is considered viable and efficient [5]. The complex nature of human health means that psychological issues often intertwine with health challenges and resolving these issues may be necessary to achieve the desired health outcomes. Pharmacists offer a listening ear to patient complaints and this makes them a critical healthcare provider. In the same vein, pharmacists relate with people that have chronic conditions that often have psychological impacts on patients and that could impact their medication use ultimately affecting their overall well-being. As a result, it is long overdue that adequate training in counseling and psychotherapy is needed to equip the twenty-first century pharmacists to meet the demands of today’s world.

With the expanding roles and responsibility for pharmacists beyond medication-related concerns comes the challenge of matching up the training of pharmacists with the broadening scope of practice in Nigeria. However, as pertinent as that might be, there are existing knowledge and competency gaps in keeping up with this trend. It has long been reported that the extant pharmacy curriculum in Nigeria is ineffective to provide adequate training as per emerging mental and public health services for pharmacy graduates, as community pharmacists do highlight inadequate knowledge as one of the barriers to their provision of certain health services such as tobacco harm reduction and social health advice [7, 8]. A study by Aluh et al. [9] reported a greater level of depression literacy among pharmacy students in comparison with non-pharmacy students in one university, however, another study found an overall negative perception and attitude towards mental illnesses among pharmacy students as comparable to non-pharmacy students in another university suggesting deficient mental health literacy [10]. Furthermore, another study found a gross level of dissatisfaction among final year pharmacy students with the adequacy of their training to offer mental health services. This study, interestingly, included as participants, students from both the Doctor of Pharmacy (PharmD) and Bachelor of Pharmacy (B.Pharm) programs—which are the two extant pharmacy curricula in Nigeria [11].

Although it is recommended and adopted that pharmacists in Nigeria engage in continuous educational programs to update their professional skills and knowledge, current evidence indicates that such personal endeavors might not yield an equally skilled population of community pharmacists especially as far as mental health services are concerned. This was underscored by Aluh and colleagues [12] in assessment of mental health literacy among pharmacists where some knowledge gaps and misconceptions about mental illnesses were found among practicing pharmacists coupled with a high level of stigma and desire for social distance, comparable to pharmacists in developed countries.

Much of the pharmacy education in Nigeria has historically centered on drug knowledge and recently, the drive for patient-centered training has the tendency to revolutionalize pharmacy training in the country. It will, however, be an incomplete revolution if the role of the social sciences is not factored into it. Humans are by nature social with a mind of their own and it is necessary that a sufficient understanding of human psychology be obtained before patients are seen and evaluated by pharmacists. This will prepare pharmacists to obtain relevant information from patients as well as use the information obtained to make better care decisions in collaboration with the patient.

## Conclusion

To address these gaps, we propose that the training curricula (the B.Pharm, Pharm.D and the Mandatory Continuous Professional Development program) for pharmacists in Nigeria be reviewed and/or expanded to provide adequate knowledge for pharmacy undergraduates and pharmacists about non-drug mental health care which will also impact on the extent of mental health services they can offer during their practice. We contend that at the very least, instruction that covers a basic introduction to understanding the human mind and behavior, relation between emotional states and physical manifestations, motivation, and mood relations to perception as well as patient assessment and communication skills be included in the revised curriculum. Modules that center on understanding psychological states for patients that belong to special populations such as drug/alcohol addicts, terminally ill patients, geriatric and pediatric patients are equally important. Stress-management techniques and an in-depth understanding of how drugs affect the mood and mind would also suffice. Such expansion of the curriculum has the potential to build a body of pharmacy professionals with requisite knowledge about mental health care than when let be for personal endeavors to compensate. Asides from providing an avenue for robust patient care, such decisive training, including the necessary referral system, will ensure quality care is provided to patients requiring mental health services in the community in order to improve mental health security in Nigeria.

## Data Availability

Not applicable.
